# Social mining for sustainable cities: thematic study of gender-based violence coverage in news articles and domestic violence in relation to COVID-19

**DOI:** 10.1007/s12652-021-03401-8

**Published:** 2022-04-08

**Authors:** Muhammad Asad Manzoor, Saeed-Ul Hassan, Amina Muazzam, Suppawong Tuarob, Raheel Nawaz

**Affiliations:** 1 Department of Computer Science, Information Technology University, 346-B, Ferozepur Road, Lahore, Pakistan; 2 Department of Computing and Mathematics, Manchester Metropolitan University, Manchester, UK; 3 Department of Applied Psychology, Lahore College for Women University, Lahore, 54000 Pakistan; 4 Faculty of Information and Communication Technology, Mahidol University, Salaya, Thailand; 5 Department of Operations, Technology, Events and Hospitality Management, Manchester Metropolitan University, Manchester, UK

**Keywords:** Social computing, SDGs, Gender-based violence, Domestic violence, Topic modeling, Word embedding, COVID-19

## Abstract

We argue that social computing and its diverse applications can contribute to the attainment of sustainable development goals (SDGs)—specifically to the SDGs concerning gender equality and empowerment of all women and girls, and to make cities and human settlements inclusive. To achieve the above goals for the sustainable growth of societies, it is crucial to study gender-based violence (GBV) in a smart city context, which is a common component of violence across socio-economic groups globally. This paper analyzes the nature of news articles reported in English newspapers of Pakistan, India, and the UK—accumulating 12,693 gender-based violence-related news articles. For the qualitative textual analysis, we employ Latent Dirichlet allocation for topic modeling and propose a Doc2Vec based word-embeddings model to classify gender-based violence-related content, called GBV2Vec. Further, by leveraging GBV2Vec, we also build an online tool that analyzes the sensitivity of Gender-based violence-related content from the textual data. We run a case study on GBV concerning COVID-19 by feeding the data collected through Google News API. Finally, we show different news reporting trends and the nature of the gender-based violence committed during the testing times of COVID-19. The approach and the toolkit that this paper proposes will be of great value to decision-makers and human rights activists, given the prompt and coordinated performance against gender-based violence in smart city context—and can contribute to the achievement of SDGs for sustainable growth of human societies.

## Introduction

Gender-based violence (GBV) is a globally widespread phenomenon and becoming an increasingly common component of violence across the world (Abdel Latif et al. 2019; Savigny 2020). According to the estimates published by the World Health Organization (WHO), one out of three women faces gender-based violence in their lifetime globally.^1^ It is assessed that ever-partnered women of age 15 years and older have been subjected to physical and/or sexual violence by their intimate partner at least once in their lifetime. These figures vary across the world, with the highest regional rates ranging from 65.64% in Central sub-Saharan Africa to the lowest 16.3% in East Asia (Palermo et al. 2014).

GBV often leads to many unpleasant public health effects, including physical disability, psychological sequelae, unwanted pregnancy, exposure to sexually transmitted infections, gynecological fistula, substance abuse and chronic pain. On top of that, there are adverse social and economic influences of GBV not only on survivors but also on their families (Ahmad et al. 2020). Proper and viable strategy reactions to address and prevent the impacts of GBV rely upon an exact and complete understanding of dynamics, prevalence, and root causes. Unfortunately, what we lack is authentic data.

In conflict situations like in the context of inadequate health care, legal and social infrastructure and especially in the case of the civil war, the magnitude of GBV remains in disguise. However, we need to know more about this in said conditions. For example, multiple sources report the rise in domestic abuses during the lockdown situation resulting in the COVID-19 pandemic. One of the important social problems is that the prevalence and pattern of GBV can only be known when there is local evidence available (Muazzam et al. 2016). Since it is largely underreported, the support programs and services could not reach too much of the potential sample and the demography of GBV remains unknown.

The reasons for this underreporting and failure to seek help are certain barriers that occur worldwide besides being considering GBV as a normal phenomenon. Many women do not seek help and report because of the threat of losing children, financial barriers, shame, and stigma, lack of awareness of available resources, perceived impunity for perpetrators, cultural beliefs, fear of getting the offender in trouble, fear of retaliation, discriminatory and stereotypical attitudes toward victims in courts and law enforcement settings, and distrust of healthcare workers (Anjum and Muazzam 2018).

There is a dearth of research available on GBV survivors’ reporting behavior in developing countries. However, over the years now, the technical reports published by NGOs active in the field repeatedly highlighted the prevalence of GBV. Largely, these reports are considered authentic because they are peer-reviewed, and they make cross-country comparisons. However, technically speaking, the prevalence and incidence of this phenomenon can largely be accessed/gauged through newspapers. This lack of data stays in the structure of the Pakistani society, which has a deep-rooted misogynistic mindset where women are ranked inferior to men. Ignorance, blind faith in clergy, and illiteracy are the prime reasons for this status quo maintained.^2^ Resultantly Pakistan stands as the 6th most dangerous country for women in the world.^3^ White Ribbon Pakistan reports that 47 034 women faced sexual violence, over 15 000 cases of honour crimes were registered, and more than 1 800 cases of domestic violence and over 5500 kidnappings of women took place between 2004 and 2016.^4^

In India, being a woman makes her vulnerable^5^ to a lot of pain, agony, and trauma as it creates a heightened risk of becoming a victim of slavery. Women face domestic abuse, child marriages, sexual harassment, and even human trafficking. India stood as the most dangerous country for women and ranked at the 1st position since the incident of gang rape and murder of a student in the bus at Delhi showed not enough was being done to tackle the danger women faced as the number of cases kept rising. According to the survey conducted by Thomas and Reuters (2018), where India was ranked 1st and Pakistan was ranked 6th, the United Kingdom was not even included in the list of the top ten countries where women were considered unsafe. Thus, we should review how their newspapers portray cases of GBV violence.

Overall, both in Pakistan and India, the GBV woman survivors face a lot of challenges in contacting legal help and police to the extent that their cases are highlighted in media sources before the judicial system picks them up. The media highlights these cases, no matter where they are from, within the country. This provides a valid reason to choose the methodology of this research for the said purpose. Newspapers are the most credible source of gathering information after social media. Most people have access to this source too. This research focused on the leading newspaper as it observed its reporting style and it is a way of communication, especially when reporting the cases involving GBV victims.

The followings are the objectives of this study:

 − **Corpus generation:** To the best of our knowledge, we find no textual data corpus available in the domain of GBV; therefore, we build our dataset using the Daily Mail from the UK, The Hindu from India, and the following three English newspapers of Pakistan (Dawn, The News, The Nation). The reason for choosing three newspapers from Pakistan is the lesser number of GBV related news articles coverage found, so to add a balance between the news articles dataset, we tried to add more news articles from Pakistan. We collected data from news articles related to gender-based violence in the past year (April 2019, April 2020). We analyze the nature of news reported in the print media, and for that, we start with topic modeling. Finally, we build our dictionary of GBV keywords by extending an existing taxonomy of GBV categories into Physical Violence, Sexual Violence and Harmful Practices.
 − **Analytical insights:** Our study consists of three portions: firstly, a comprehensive comparison study on GBV news articles from all newspapers was conducted. In this part, we evaluate the nature of topics discussed while reporting news related to gender-based violence. Secondly, we study the composition of these topics, like keywords most used in these topics and their relevance. Later on, we discuss the division of topics across news articles, i.e., the number of documents by topic weightage against each topic. In the final step, a third dataset is built using all the news articles which contain any of the keywords from the GBV dictionary. After data cleaning and preprocessing, topic modeling is applied to the dataset, and then topics covered by the dataset are compared. After topic comparison, GBV keywords coverage is compared across the dataset. Finally, we present a comparative study using the newspaper Daily Mail, The Hindu, and The News using one-year news articles data from April 2019 to April 2020. We analyzed the themes of the reported news and topics discussed in a dataset containing GBV related news articles of the newspapers in a given period across the nations, UK, Pakistan and India.
 − **GBVMeter for COVID-19:** To analyze textual content concerning GBV, we build a GBVMeter tool that takes unstructured text as input from any online source such as blog, news or website and output the intensity of GBV related content along with the prominent topics and terms within GBV. This tool leverages GBV2Vec for real-time analysis of web content, i.e., a Doc2Vec based word-embeddings trained specifically to classify GBV textual content. Finally, we employ a use case to understand the impact of COVID-19 concerning GBV. We utilized Google News API to fetch top trending global news against the following search query: “Gender-based Violence and COVID-19”. Finally, the results are displayed on a dashboard using our GBVMeter tool.

The rest of the paper is structured as follows: Sect. 2 presents a literature review. Section 3 presents the data collection steps and employed approaches. Section 4 presents experiments and results. Finally, conclusions have been discussed in Sect. 5.

## Literature review

In this section, we discussed the framework for the quantitative and qualitative study of GBV. The goal is to identify previous work in this domain and support our research using already explored work. We have divided the literature review into three parts. (i) Gender-based violence, (ii) topic modeling, (iii) text analysis.

In the first part of “gender-based violence,” we have studied previous work done on gender-based violence and gender bias identification reported in the news. Unfortunately, we find only a handful of studies in this direction, including Brooks and Hayes (2019), who studied the effects of political campaigns through a survey. The authors concluded that female candidates’ support would be increased if they are subjected to gender bias violence. Leavy (2019) performed a comprehensive study on coverage of male and female politicians in new papers to identify potential gender bias. Toffoletti (2007) studied the behavior and narrative of stories of violence against women in Australian newspapers. They analyzed that media institutions can influence the understanding of gender and public discourse. Rheault et al. (2019) studied the nature of abuses and derogatory comments faced by legislators over social media using machine learning unsupervised techniques.

Buiten and Salo (2007) studied the reporting of GBV in newspapers. They analyzed the role of media in construction and challenging the current state of gender relations, i.e., representation of women in different sections of media. Kangaspunta and Marshall (2012) discussed the internationally agreed-upon definition of “violence against women” adopted in the United Nations General Assembly Declaration. Boonzaier (2017) discussed the need to study the representation of sexual and gendered violence in a historical context. Tranchese and Zollo (2013) conducted a comparative analysis of perpetrators of rape and victim’s representation in the broadcast and printed media. Purohit et al. (2015) analyzed public sentiment linked to gender-based violence by analyzing the Twitter dataset. Moss-Racusin et al. (2012) performed a case study of rating job applications by science faculty from top research institutions. They found that male-named applications were rated more and considered more appropriate for the position. Boring (2017) used a French dataset to evaluate gender-biased evaluations of teachers by students. These studies conclude that gender-based violence (GBV) is a globally widespread phenomenon and becoming an increasingly common violence component across the world. It is assessed that ever-partnered women of age 15 years and older have been subjected to physical and/or sexual violence by their intimate partner at least once in their lifetime. GBV often leads to many unpleasant public health effects, including physical disability, psychological sequelae, unwanted pregnancy, exposure to sexually transmitted infections, gynecological fistula, substance abuse, and chronic pain.

Helmer et al. (2017) studied the representation of women in the peer-review method. They found that women are less represented in the peer review process and both gendered editors have the same choices overall. Zhao et al. (2018) proposed new standards for coreference resolution concentrated on gender bias called WinoBias. The authors presented a general-purpose system for performing coreference resolution. Baker (2013) studied incidence and situations in which gender-pointed expressions are used and found that gender-specific conventions reduce over time. Lawrence (2013) suggest that women political candidates face challenges in presenting themselves to voters. Media campaigns affect how voters judge candidates. Vickery and Everbach (2018) studied cloverleaf of gender, technology, and media and conducted a study to analyze the nature and situations of harassment faced by females. Sikweyiya and Jewkes (2011) built a dataset using interviews with twelve experienced gender-based violence researchers from different countries. The authors concluded that observational evidence is needed to support the affirmations of uncertainty in research.

In the second part, “topic modeling,” we studied and explored various methods to build topic modeling using LDA. Blei et al. (2003) proposed a generative probabilistic model for collections of discrete data. Mehrotra et al. (2013) proposed different methodologies to improve the performance of topic identification using the LDA model. Lim et al. (2017) proposed a methodology to automatically identify the discussion topics without any external support and parameter-based tunings and other techniques. Hong and Davison (2010) proposed a technique to understand the hidden meaning in the text. This technique is utilized to identify various conceptual entities in text, e.g., important news, potential threats, people’s opinion, etc. Kim and Gil (2019) proposed a technique to classify research text material and papers into a standard set of classes based on the nature of topics discussed in them.

Hoffman et al. (2010) proposed a technique to perform online topic modeling using LDA. Tong and Zhang (2016) represent an introduction to text mining and Latent Dirichlet allocation. Multiple experiments are also proposed. The experiment process includes data collection, data preprocessing, and model training. Pavlinek and Podgorelec (2017) proposed a text classification technique for a small dataset. This technique is based on self-learning topic modeling. Kiritchenko and Mohammad (2018) proposed the equity evaluation corpus consisting of more than eight thousand English sentences. The authors found that out of all the available sentiment analysis systems, they are mostly significantly biased.

In the next part, “text analysis,” we explored text classification approaches and methodologies applied. Le and Mikolov (2014) proposed an unsupervised algorithm, “Paragraph Vector.” This algorithm provided the best results on sentiment analysis and text classification. Lee et al. (2011) distributed Twitter trending topics into eighteen comprehensive categories. The authors applied two methods for topic classification. Eliacik and Erdogan (2018) proposed a technique for sentiment analysis. The methodology examines social account information for sentiment analysis as well. Zhu and Hu (2017) proposed a modification to the Doc2Vec algorithm. Hoque et al. (2019) trained a model using a dataset built using more than seven thousand Bengali language sentences and compared their accuracy to classify data. Zhang and Baldwin (2019) proposed an extension to word-level embeddings, extended the scope to the complete document, and studied multi-relational classification. Mikolov et al. (2013) proposed two new model architectures for the computation of vector illustration of words. The authors identified that these vectors produced the best performance on semantic and syntactic word similarities measuring tests.

Bolukbasi et al. (2016) performed a series of experiments to conclude that blind applications of machine learning can lead to increased biases in data. Dixon et al. (2018) proposed a new methodology to reduce bias in machine learning models. The authors balanced the training datasets and applied unsupervised methods to minimize bias. Finally, Garg et al. (2018) proposed a new architecture to understand gender-specific changes in attitude and stereotypes affected by embedding and studied the impact of embedding on attitudes towards minorities.

## Data and experimentations

In this section, we discuss design techniques utilized to study the behavior of news articles while reporting about females and gender-based violence. First, we show the composition of the dataset and the different subsets of the dataset that were produced for our experiments. Then, we discuss the preprocessing of our dataset and the various tools and technologies used for performing research work. We build a dictionary of GBV keywords, which are used to identify potential GBV related articles. Next, we formulate a dataset for our study, comprising news articles of prominent English newspapers of Pakistan, England, and India utilizing GBV keywords extracted during 2020. The newspapers include The News, Nation, Dawn from Pakistan, Daily Mail from the UK, and The Hindu from India. Next, we build a dictionary of gender-based violence terms, as shown in Table 1, by extending existing keywords (Purohit et al. 2015). Then, we filtered GBV news articles using this dictionary. Finally, the GBV keywords containing news articles are used for topic modeling and word embedding.

**Table 1:** GBV News dataset with source newspapers

Source	# of news articles
Daily Mail	8795
The News	348
The Nation	440
Dawn	169
The Hindu	2941

### Data preprocessing

We build a data set by scraping e-newspapers. We use a scrappy framework for building spiders to scrap news article data. In this step, the main challenge is to extract clean and valuable information against each news article. In the text preprocessing step, we remove stop words using NLTK^6^ library and use Spacy’s^7^ English model for text lemmatization. The lemmatization converts words into their base forms. For example, ‘houses’ is lemmatized into ‘house.’ We utilize MongoDB for storing datasets. Further, we import that data and tokenize it. Here, every sentence is divided into a collection of words and cleaned by removing emails, newline characters, single quotes, punctuations, and other unnecessary characters using regular expressions.

### Approaches

In this subsection, we discuss the employed approaches to analyze news reporting related to gender-based violence using topic modeling. In the next section, we present the textual analysis of our data using GBV2Vec models. Finally, we present the tool that we built using GBV2Vec models to classify gender-based violence-related content from unstructured text.

#### Topic modeling

Topic Modeling is used to extract the hidden meanings and semantic structures from significant volumes of text. Instinctively, a text or document is about any topic—it is understood and expected behavior that a certain set of keywords will appear in that text frequently. For topics related to gender violence, it will be understood that keywords like violence, gender-specific keywords, different forms of violence, i.e., violence against women, and other similar keywords may exist in the document. There is a possibility that more than one topic exists in a document, whereas the quantity of these topics can vary.

Topic models are called probabilistic in nature. The model explores the set of text documents and identifies the hidden topic of the text document and balance of multiple topics in it, using the statistics of the words in each document. We have used the latent Dirichlet allocation (LDA) algorithm, which is one of the most widely-used topic modeling algorithms in the information retrieval methodology. This algorithm has great implementations available. Moreover, it is known to run faster and gives better topic segregation.

**Fig. 1:**
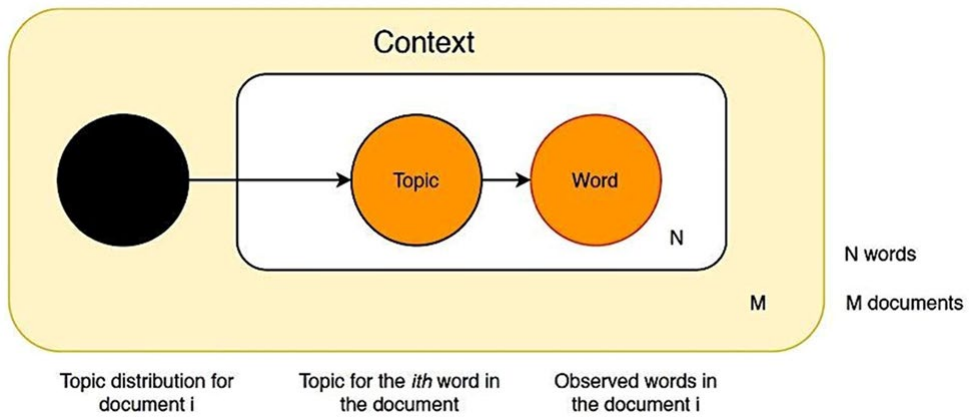
Basic workflow of LDA topic modeling

LDA is an unsupervised machine learning model that considers each text as a bag of words. The LDA hypothesizes that each text document is a set of topics. In each topic, there is a set of words related to that topic, as shown in Fig. 1. LDA starts working by building a set of topics against each document. The algorithm distributes topics across all the documents. Each topic is assigned a word. The word shows that it is related to that topic. The relation of words with a topic is explored and defined based on the document's topics and how many times that specific word is assigned to the document and overall across all the documents. The algorithm repeats this process several times against each document before settling down with final decided topics against each document. LDA output contains all the topics made of all the words with their probabilities to belong to a topic.

After data preprocessing, we form bigrams and trigrams. A bigram is two consecutive words. Similarly, the trigram is three frequently occurring words. We prepared a dictionary and a corpus for our model. Each document is mapped to a unique id. Now we train our LDA model on top of it. Finally, we tune hyperparameters affecting the sparsity of the topics.

We used the following parameters to tune our model.

- **Alpha = 2000**—hyperparameter affecting the sparsity of the topics.
- **Chunksize = 10**—number of documents to be used in each training chunk. We use ten documents to train in each training chunk.
- **Update_every = 1**—number of documents to be iterated through for each update. We set it equal to 1 for iterative online learning.
- **passes = 10**—number of passes through the corpus during training. We used ten training passes for our model.
- **Iterations = 100**—maximum number of iterations through the corpus when inferring the topic distribution of a corpus. We did 100 iterations for this process.

A topic is a combination of keywords and their contribution to a topic in terms of probability mixture. Our LDA model is built with four different topics. Initially, we tried six and eight topics for models, but we identified that topics start repeating after four topics. After getting topics, we perform a series of qualitative analyses on these topics. We discuss more of these analyses in the experimental results section.

#### Text classification with GBV2Vec

**Fig. 2:**
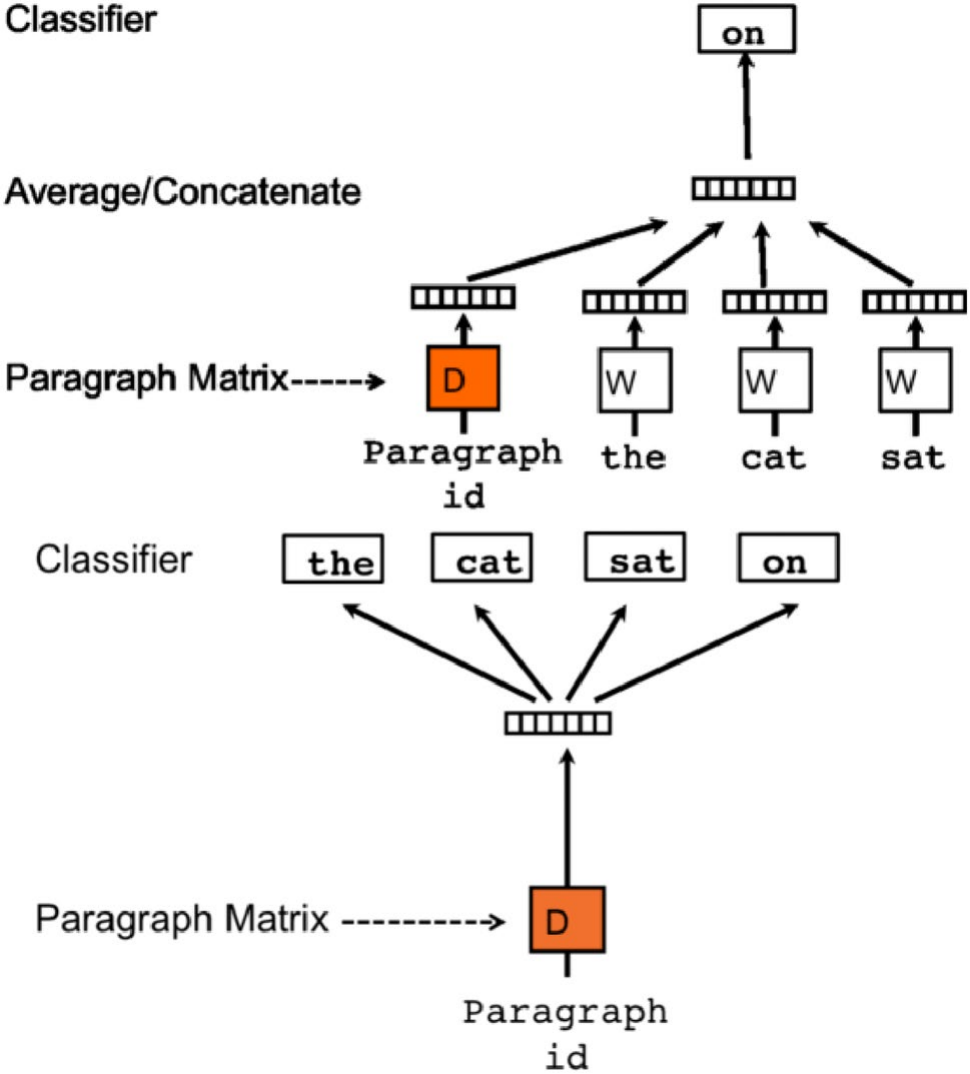
Architecture of (left) PV-DM, PV-DBOW (right) (Le and Mikolov 2014)

Our proposed GBV2Vec is a machine learning algorithm based on Doc2Vec (Le and Mikolov 2014), that is used to represent text documents in the form of vectors. GBV2Vec is an unsupervised learning method to understand document representation by inputting documents with flexible size in terms of words per document, but the output is fixed-length vectors. In GBV2Vec architecture, there are two algorithms, “distributed memory” (PV-DM) and “distributed bag of words” (PV-DBOW), as shown in Fig. 2.

In distributed memory, paragraph and word vectors are initialized, where each paragraph vector is assigned a document and word vectors are shared across all documents. This methodology is an extension of the continuous bag of words. A paragraph vector is used to classify entire words in documents in a distributed bag of words algorithm. This approach is an extension of the skip-gram algorithm. Distributed bag of words algorithm builds a classifier to identify word and document relations and possibly help in mapping words to their appropriate documents.

We use multiprocessing for faster training with GBV2Vec. The dataset prepared for the LDA model will be used while creating training and testing documents as input to the GBV2Vec model. The dataset has 12,693 news articles, comprising randomly selected 11,000 news articles for training. The remaining news articles are reserved for testing. In the next step, we tagged each document with a token as the first argument and label ID as the second argument (5: violence against women) for Tagged-Document. Next, we trained the Logistic Regression Classifier (LRC). The LRC is a supervised machine-learning model used to detect the possibility of the existence of a certain class. It is used for text classification. We used the feature vector generated for the train documents while training and used the feature vectors of test documents in the prediction phase.

Further, we evaluate the results of models trained using GBV2Vec’s distributed memory and distributed-bag-of-words algorithms. These models are trained on news articles that have content related to gender-based violence. Using these learned models, we can now find the similarity of provided text with gender-based violence-related content. In the distributed memory or DM model, the central word is predicted using the context information from the set of input words, giving an accuracy of 53%. On the other hand, the distributed-bag-of-words model neglects the context of input words and predicts words randomly from the text document. Therefore, its performance was better, with an accuracy of 67%.

#### GBVMeter content analyzer tool

A web portal is also built (called GBVMeter) as a working prototype on top of the model trained in GBV2Vec for real-time web content analysis. A GBVMeter is a web portal that provides the two main functionalities. Any web page content can be analyzed by submitting an address (URL) of the web page, and a dashboard will be built on top of the content of that web page. Users can provide the address (URL) of any website, portal, blog, or News (Application Programmable Interface) API to this web portal to analyze the content on that page and build a dashboard with detailed analysis.

The dashboard is created using a trained machine learning model after analyzing the content of the dataset furthermore web pages. Four reports are part of the dashboard. The first report shows a GBV content matching percentage meter. This meter represents the accuracy of content matched to that of GBV based models trained. The second report shows word cloud representation of the content, identifying major keywords involved in topics. The third and fourth reports represent the coverage of the content across GBV categories and keywords (as shown in Table 2.)

**Table 2:** GBV terms dictionary divided into categories

Category	GBV terms
Physical violence	Girl burn, women beaten, woman dragged, woman kicked, woman beat up, women burn, woman acid attack, woman violence, domestic violence, female beaten, women dragged, women kicked, women beat up, women acid attack, women violence, violence against women, woman beaten, girl dragged, girl kicked, woman burn, girl beat up, girl acid attack, girl violence, domestic abuse, female dragged, female kicked, female beat up, female acid attack, female violence, girl beaten, female burn
Sexual violence	Sexual harassment, woman harass, woman attacked, boyfriend assault, stalking woman, groping woman, sexual assault, women harass, women attacked, boy-friend assault, stalking women, groping women, sexual violence, girl harass, girl attacked, stalking girl, groping girl, rape, female harass, female attacked, stalking female, groping female
Harmful practices	Female trafficking, child marriage, forced marriage, woman trafficking, children trafficking, children marriage, women trafficking, sex trafficking, child trafficking, underage marriage, girl trafficking

## Results and discussion

To analyze GBV related content in the selected newspapers, we perform a series of experiments. Initially, a topic modeling using LDA is applied to analyze the topics reported in the media using a dataset consisting of major English newspapers of Pakistan (The News, Nation, Dawn), Daily Mail from UK and The Hindu from India. We build a dictionary of gender-based violence keywords by extending existing keywords (Purohit et al. 2015). Then we train the machine learning model to incorporate it into a web portal to analyze web content based on GBV. An overall analysis of this dataset was done using different visualization techniques, and different reports are generated to complete our case studies.

### GBV topics in news articles

**Fig. 3:**
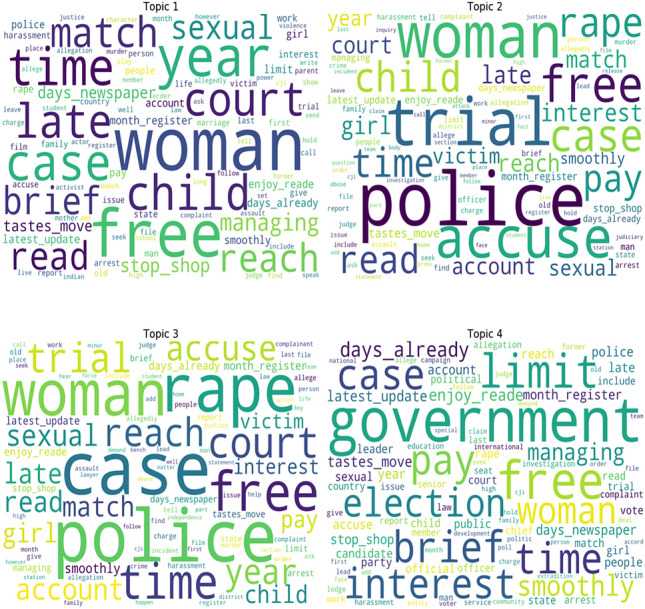
Dominant GBV topics from LDA analysis

In this section, we utilized the dataset of all newspaper articles and built a corpse for the LDA model and analyzed topics covered by newspapers. We selected the top 4 topics using LDA. Our analysis as shown in Fig. 3 found that the most discussed topic about GBV was a rape case, sexual assault, and domestic abuse. In the first topic (Topic 1) news around sexual assault, court hearing, and violence against women and children are reported. More empirical analysis of the news collected in this clusters shows the involvement of court in women rape cases.

The second topic (Topic 2) showed more specific news reporting related to GBV, like trial proceedings, police point of view and discussed the nature of cases and victims. The third topic (Topic 3) news is about reporting the casing against minors and especially against girls. Police actions to eliminate child rape cases and perform an investigation of child rape cases are reported.

In the fourth topic (Topic 4), government and police efforts to curb violence against women, but rape cases can still be seen. The empirical analysis on the news associated with this topic shows the discussion of government performance in reducing gender-based violence is observed. Also, the stories related to government efforts to stop gender-based violence are reported. In addition, news around sexual abuse and assault by politicians and celebrities is also widely reported, especially during election campaigns.

**Fig. 4:**
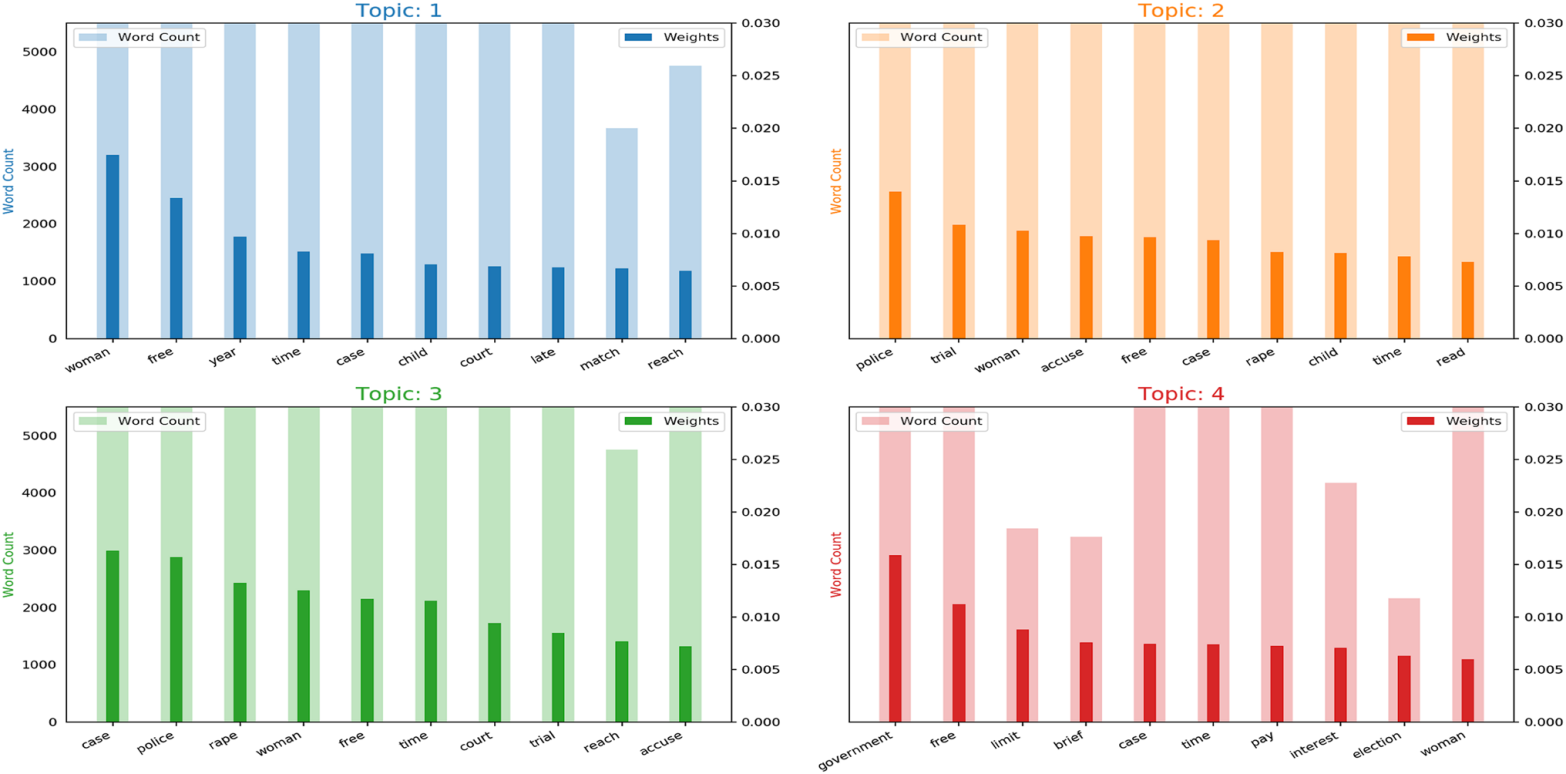
Word count and importance of topic terms against each topic

Further, these topics are analyzed to understand the division of words composing these topics and their importance. Topics selected by the LDA model are studied in more detail, where each topic is analyzed to identify the keywords consisting of these topics and analyze the weightage of these words and understand the impact of these keywords in depicting the topic name, as shown in Fig. 4.

**Fig. 5:**
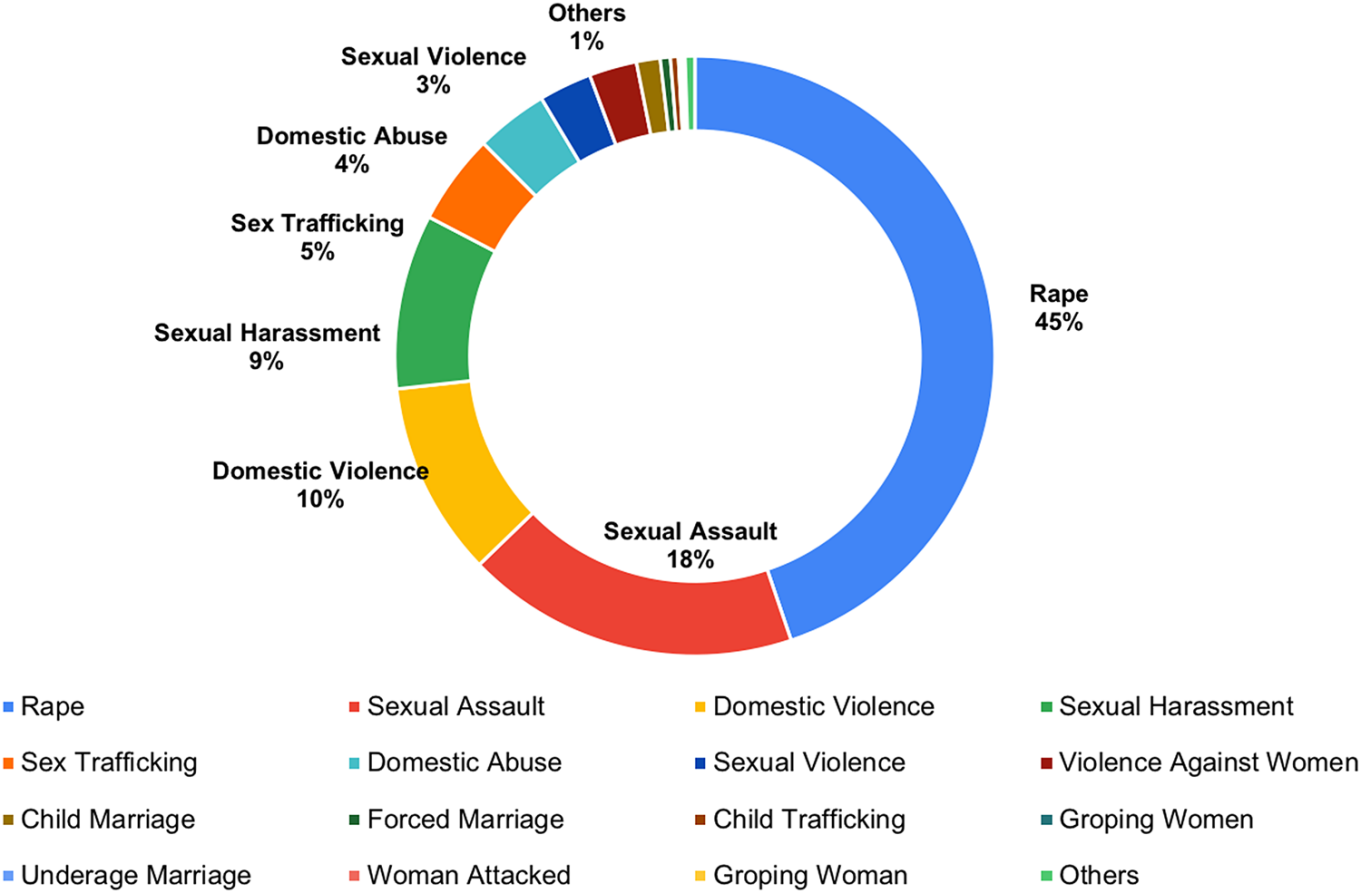
Top 15 GBV terms in dataset

Further, mapping the news across the GBV categories with Table 2, we analyzed that “woman” and “rape” are among the most used keywords in the topics that are based on the reporting of gender-based violence-related news. This shows that women are the main victim of gender-based violence in most of the news in our collected dataset (see Fig. 5). In addition, we found that rape cases are the most reported gender-based violence. This topic is dominant across the majority of news articles. Apart from the raping topic, domestic violence, violence against women, and sexual violence are the other main topics discussed in the news articles.

### Comparative study of Daily Mail, The Hindu, The News

A comparative analysis has been done using news articles data from “Daily Mail”, “The Hindu” and “The News” from April 2019 to April 2020. In this study, we show distribution of news articles within subcategories or keywords of GBV.

**Fig. 6:**
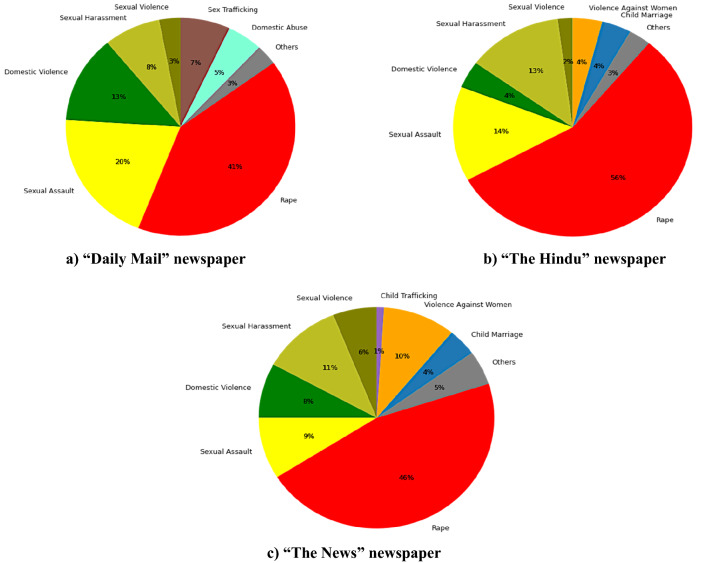
Dominant GBV Terms in **a** Daily Mail, **b** The Hindu, and **c** The News

As shown in Fig. 6 the Daily Mail newspaper coverage concerning to the GBV news is more diverse and cater verity of the aspect of GBV like domestic abuse, sexual assaults, violence against women. The Hindu (India) and The News (Pakistan) have almost similar pattern of reporting and we identify that the most news is related to sexual abuse and rape cases. In Pakistan cases of child abuse are also concerning and that is also highlighted in news articles.

Violence against women, especially rape, is the most discussed topic. The Hindu from India has a major share of news related to rape due to an increasing number of rape, sexual harassment, and sexual assault crimes against women. Overall, India and Pakistan show more concentration of rape-related news as compared to the UK in our dataset.

### GBVMeter for Google News analysis in relation to COVID-19

By leveraging the GBVMeter, we showcase a study to analyze the impact of COVID-19 in relation to GBV. We have utilized Google news API to fetch the top 100 trending news against the search terms “Gender-based Violence and COVID-19”—from April 15, 2020, to May 15, 2020.

**Fig. 7:**
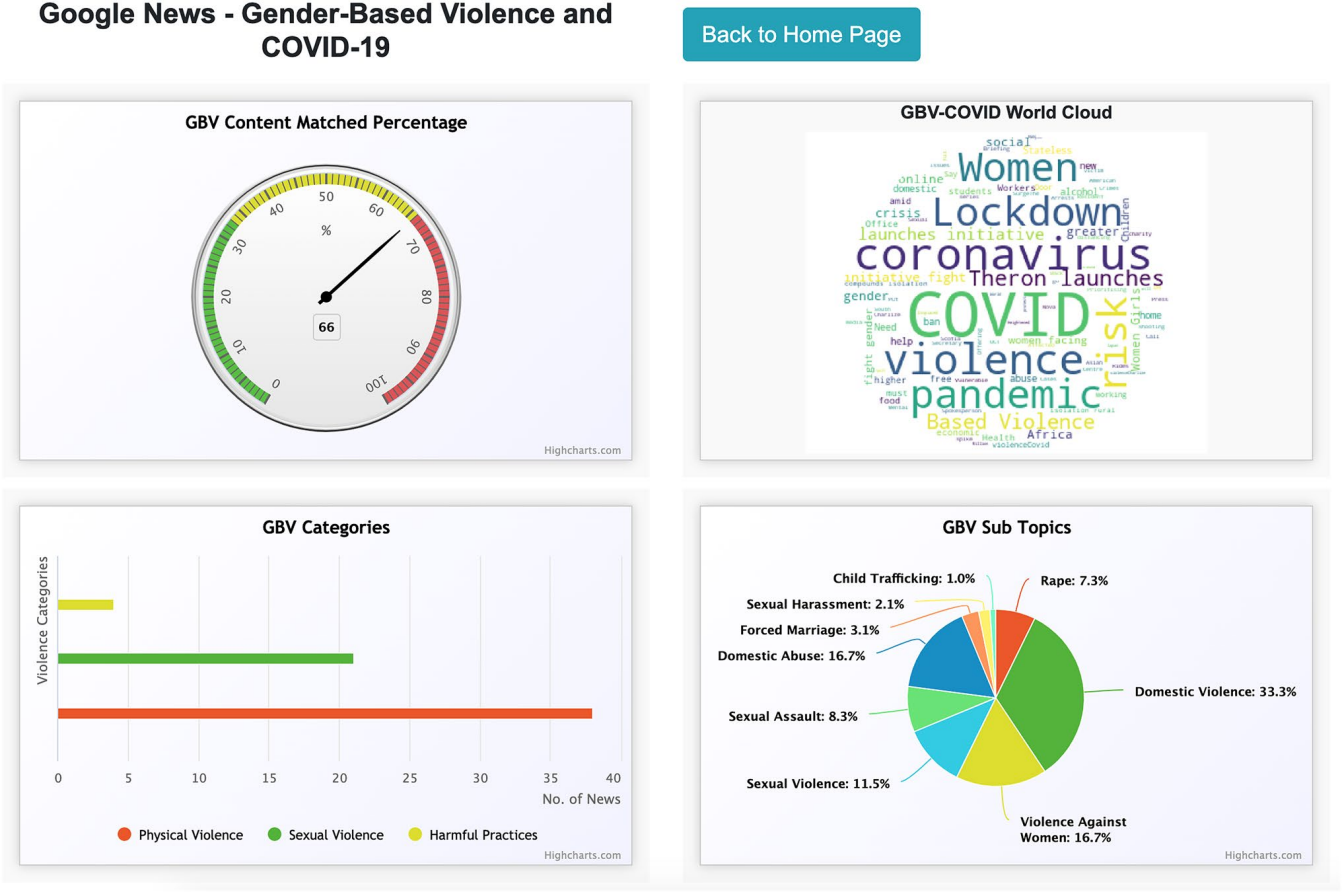
GBVMeter analytics dashboard for “Gender-based Violence and COVID-19” search during April 15, 2020 to May 15, 2020

Using this dataset, we build an analytical GBVMeter dashboard (see Fig. 7). The dashboard consists of the following four components: the first one is the GBV content meter which shows the sensitivity of GBV related content in the input text as classified by the pre-trained GBV2Vec model. Since the input text in this case study is pertaining to GBV in relation to COVID-19, we find high sensitivity of GBV related content, i.e., up to 70%.

The second component is the word cloud—comprising the most frequent terms from the text. We find the following terms are the most prominent in the selected dataset of news: women, violence, coronavirus, lockdown, pandemic etc. We also find the keyword ‘Charlize Theron,’ which is pertaining to the campaign launched by the actress by donating half a million US dollars to fight gender-based violence during COVID-19. The third component shows the division of news data across GBV categories. We find that physical violence is more reported gender-based violence in recent times, with over 35% of news fall into the category of physical violence followed by sexual violence with around 20%.

The fourth component helps us to understand the topics discussed in the news in more detail. We find that domestics violence, violence against women, sexual assault, and sexual violence are the most frequent terms. We also find rape and child trafficking related terms; however, their presence is relatively low compared to the subtopics that fall under physical violence (see Table 2). Our analysis shows that the COVID-19 pandemic has increased in physical violence.

## Concluding remarks

This paper showcases the ways social computing and its diverse applications can contribute to the attainment of SDGs for the sustainable growth of human societies. More specifically, to the SGDs concerning to make cities and human settlements inclusive, safe, resilient, and sustainable, and gender equality and empowerment of all women and girls. By employing state-of-the-art computing methods, we study the state of GBV in a smart city context. We find that adequate coverage is extended to all leading newspapers related to GBV in Pakistan, India and the UK, focusing on physical violence, sexual violence and harmful practices, with rape being the most reported crime. However, solutions to these ill practices are not offered in the news coming from the subcontinent. It is essential to report that the heinous crime of rape and related news is published mainly in Pakistan and India.

We also show that the COVID-19 pandemic has increased the prevalence of GBV across the globe; women were the prime victims. More research is needed to offer detailed insight into the scale of GBV as it unfolded during COVID-19 inflicted lockdown around the world. Research on Covid-19 and its diverse social and political ramifications gradually reaches the mainstream academic debate (Visvizi and Lytras 2020; AI-Youbi et al. 2020). Nevertheless, more detailed insights are needed to understand the specific role governments, media (including social media) and other stakeholders should play in times of pandemics. The key question in this context is how to mitigate adverse social implications of measures, like lockdown, undertaken to limit the spread of COVID-19. Our future work will enhance the accuracy of models by improving dataset size and quality (Zhang et al. 2018). Apart from the LDA model and GBV2Vec, we will explore other machine learning algorithms to improve the employed models' accuracy (Borkar et al. 2019).

## Footnotes

1 https://www.who.int/news-room/fact-sheets/detail/violence-against-women.

2 http://uis.unesco.org/en/country/pk.

3 https://poll2018.trust.org/country/?id=pakistan.

4 http://www.whiteribbon.org.pk/understand-domestic-violence/statistics-of-vaw/.

5 https://poll2018.trust.org/country/?id=india.

6 https://www.nltk.org.

7 https://spacy.io.
